# Health-related quality of life in patients undergoing hepato-pancreato biliary cancer surgery: A prospective follow-up study

**DOI:** 10.1016/j.sopen.2025.06.008

**Published:** 2025-06-20

**Authors:** Anna Ekström, Victoria Fomichov, Bergthor Björnsson, Carina Wennerholm, Per Sandström, Jenny Drott

**Affiliations:** a Division of Nursing Science and Reproductive Health, Department of Health, Medicine and Caring Sciences, Linköping University, Linköping, Sweden; b Unit for Public Health and Statistics, County Council of Östergötland, Linköping University, Linköping, Sweden; c Department of Surgery in Linköping, Department of Biomedical and Clinical Sciences, Linköping University, Linköping, Sweden; d Linköping Comprehensive Cancer Center, Linköping, Sweden

**Keywords:** Cancer, Health, Health-related quality of life, Surgical care, Patient-reported outcomes

## Abstract

**Purpose:**

Patients with cancer in the liver, pancreas, or bile ducts often experience stressful situations. These patients frequently perceive a variety of symptoms that affect their health both before and after surgery. This study aimed to investigate the health-related quality of life of patients undergoing hepato-pancreato-biliary cancer surgery and to compare pre- and postoperative patient-reported outcomes.

**Method:**

A prospective study was conducted using a consecutive sampling procedure. The inclusion criteria were patients aged over 18 years with malignant tumours in the liver, bile ducts, or pancreas, who were treated with curative cancer surgery.

**Results:**

Of the 77 included patients, 50.6 % were men, and 55.8 % were 70 years or older. The results are based on 154 completed EQ-5D-5L questionnaires, analyzed preoperatively and postoperatively. The overall mobility and anxiety dimensions did not change between the pre- and postoperative assessments. The overall self-care dimension increased over time (*p* = 0.001), as did the usual activities and pain dimensions (*p* < 0.001). More men reported no problems with mobility one month postoperatively. Our results showed that patients undergoing HPB cancer surgery had a lower overall health-related quality of life postoperatively, with significant differences in the dimensions of pain, activity, and self-care.

**Conclusions:**

Clinically significant results include no changes in mobility and anxiety between the pre- and postoperative assessments. Men reported higher rates of no problems with mobility postoperatively. The differences in mobility between women and men are important clinical findings, suggesting the need for more attention to support patients for equitable and safe postoperative cancer care.

## Highlights

• Ensure that quality of life is followed up with measurements even in a shorter time perspective after HPB cancer surgery.
• There may be gender differences in quality of life, which may entail adapted supportive interventions based on graded quality of life postoperatively.
• The findings suggest a need for more attention to support patients, ensuring equitable and safe postoperative HPB cancer care.

## Background

Patients with cancer in the liver, pancreas or bile ducts are a vulnerable patient group with a stressful and tough situation. The patients often perceived a variety of symptoms affecting their health-related quality of life (HRQoL) both prior to surgery and in the postoperative period [1]. Cancer surgery is a cause of great stress for the body, and it can take time before the patient begins to feel fully recovered after the surgery. Major surgeries are associated with nausea, pain, anxiety, and postoperative complications [2]. Therefore, it is often presumed to substantially reduce HRQoL, at least in the early postoperative phase. Previous evidence has shown that patients with cancer in the liver, pancreas or bile ducts have low survival rates, and the diagnosis and treatments affected the patients' HRQoL. Cognitive and mental changes and coping with the disease are common for patients with this type of cancer [1,3]. Currently, short hospital stays are common, which means that patients need to perform self-care to cope with their situation at home [4].

Patient-reported outcome measures (PROMs) are important to receive patients' views of their health in cancer care and of importance for cancer nursing. ESMO Guidelines recommend PROMs during the care of patients with cancer from the start of active treatment and during the treatment trajectory [5]. By using a validated HRQoL questionnaire and individualized care based on the individual answers from the patients, previous research has shown positive outcomes [6]. To our knowledge, follow-up studies in patients with malignant tumours in the liver, pancreas, or bile ducts are sparse due to their perceived HRQoL one-month post-surgery. The study aimed to investigate the HRQoL of patients with hepato-pancreato-biliary (HPB) cancer surgery and compare pre- and postoperative patient reported outcomes.

Research questions:

• Are there any differences between pre- and one-month postoperative regarding the EuroQol-5 Dimensions?
• What change is of importance for clinical surgical cancer care, regarding EQ-5D-Index and EQ-5D-VAS (perceived health) pre- and one-month postoperatively?

## Methods

### Design

A prospective study was conducted, guided by the STROBE Statement and the checklist of cohort studies [7].

### Participants, setting and data collection

The patients were recruited from one university hospital in southeast Sweden. A consecutive sampling procedure was used for the inclusion of patients who underwent surgery for upper abdominal cancer in a specialist surgery clinic between September 2019 and April 2020.

The inclusion criteria were patients >18 years with malignant tumours in the liver, bile ducts or pancreas treated with curative cancer surgery. The exclusion criteria encompassed patients with cognitive impairment or those lacking proficiency in the Swedish language, as it was imperative to complete the questionnaires in Swedish. Furthermore, patients deemed inoperable for diagnosed HPB cancer, as assessed by a surgeon, were excluded. All patients were routinely advised to cease smoking and alcohol consumption prior to surgery. This is in accordance with standard care and current clinical guidelines. They possessed a general condition conducive to surgical intervention and were considered capable of enduring surgery for the tumour disease.

The included patients completed the EQ5D questionnaire preoperatively. The patient answered the questionnaire either during the pre-surgery appointment visit at the clinic or completed the questionnaire at home and brought it with them when they were admitted to the hospital the day before the elective surgery. The patients completed the questionnaire between 1 and 7 days prior to undergoing elective surgery. This preoperative timeframe was chosen to ensure that patients had sufficient opportunity to provide accurate and thoughtful responses while still being close enough to the surgery date to reflect their pre-operative condition. By administering the questionnaire within this period, we aimed to capture the patients' health status and concerns effectively, which would contribute to a comprehensive understanding of their pre-operative baseline.

The post-operative questionnaire was completed 2 weeks after the patients were discharged from the hospital. The patients received the questionnaire upon discharge and were instructed to complete it at a specified time, which was scheduled for 2 weeks after the surgery. This time point was selected to provide patients with adequate recovery time before they were asked to give feedback on their post-operative health condition. If patients did not respond to the questionnaire within 2 weeks, follow-up reminders were sent to encourage completion. These reminders aimed to ensure a high response rate and to collect comprehensive data on the patients' health outcomes.

A total of 101 patients provided both oral and written informed consent to participate in the study. Of these, 77 patients successfully completed both the pre-operative and post-operative EQ5D questionnaires. However, 24 patients did not return the post-operative questionnaire, resulting in a response rate of 76.2 %. This response rate reflects the proportion of patients who provided comprehensive data for both time points, which is crucial for evaluating the study's aim, outcomes and ensuring the reliability of the findings.

### Measures

The Swedish validated version questionnaire EQ-5D-5 L was used in the study. The EQ-5D-5 L consists of a patient-reported descriptive system/assessment and the EQ Visual Analogue scale (EQ VAS). The EQ-5D-5 L descriptive system comprises the following 5 dimensions: mobility, self-care, usual activities, pain/discomfort, and anxiety/depression. The descriptive system/assessment comprises five levels: no problems (Level 1), slight problems, moderate problems, severe problems, and extreme problems (Level 5). A unique health state is defined by combining 1 level from each of the 5 dimensions. EQ-5D-5 L health states, defined by the EQ-5D-5 L descriptive system, can be converted into an index [8]. The index values, presented in country-specific value sets and the Swedish value set, were used in this study [9]. The EQ VAS was used to determine the patient's self-reported general health status on a scale of 0 to 100. A score of 100 represented “The best health status I can imagine,” whereas 0 represented “The worst health status I can imagine.”

### Ethics

The study was performed in accordance with the ethical principles of the Declaration of Helsinki [10] and approved by the Regional Ethics Review Board (No. 2016/276–31).

### Statistical analysis

Descriptive statistics were used to present patient characteristics and demographics, as well as for the patients' reported EQ-5D-5 L (dimensions, index score and EQ-VAS before and after surgery). Proportions and counts were used for all except the Index Score and EQ-VAS, which were calculated as the mean and standard deviation for each time point.

Repeated Measures Analysis of Covariance (ANCOVA) was carried out to further study EQ-VAS and EQ-5D Index Score changes between preoperative and postoperative. Likewise, to study the changes in the EQ-5D-5 L dimensions, a repeated measures multivariate analysis of covariance (MANCOVA) was used due to the multiple dependencies among them. Each dimension was dichotomized to a dummy where “No problems” received the value 1. The models estimated the means, and their respective 95 % confidence intervals adjusted for relevant covariates. Differences between time points were tested, and *P* values lower than 0.05 were considered significant. The analysis was performed for the total study population and split by sex and tumour. The results from patients with bile duct cancer were not presented due to great uncertainty caused by the low number of observations.

Analysis of variance (ANOVA) was also performed for the mean differences between time points of the EQ-5D Index Score and the EQ-VAS for the variables sex, age, children, education level, marital status and tumour. The models did not find any significant differences within the variables; therefore, no further analyses were made to compare the adjusted results for each variable. These analyses are not shown. All analyses were performed in IBM SPSS Statistics 27 (SPSS Inc., Chicago, IL, USA).

## Results

The included patients in the study, *n* = 77 (50.6 % were men). A total of 154 answered EQ-5D-5 L questionnaires were returned pre- and postoperatively and were analyzed (response rate 76.2 %). In addition, 55.8 % were 70 years or older, nearly all patients are over 60 years (87 %). The surgical interventions for tumours encompass pancreaticoduodenectomy (22.1 %), distal pancreatectomy (10.4 %), total pancreatectomy (2.6 %), major liver resection (42.9 %), and minor liver resection (22.1 %), specifically addressing malignancies of the pancreas, liver (hepatocellular carcinoma, metastatic colorectal cancer), and bile duct (cholangiocarcinoma).

Details regarding patient demographics are shown in Table 1.

**Table 1:** Patient characteristics and demographics.

		Percent (n)		
Sex	Female	49.4 %	(38)	
	Male	50.6 %	(39)	
Age	30–39	1.3 %	(1)	
	40–49	2.6 %	(2)	
	50–59	9.1 %	(7)	
	60–69	31.2 %	(24)	
	70-	55.8 %	(43)	
Education level	Compulsory school (through grade 9)	35.1 %	(26)	
	2 years high school, trade school	10.8 %	(8)	
	High school, 3–4 years	24.3 %	(18)	
	University, college	24.3 %	(18)	
	Other	5.4 %	(4)	
	*Missing*			*3*
Marital status	Married/partner	76.6 %	(59)	
	Single	20.8 %	(16)	
	Other	2.6 %	(2)	
Children	Yes	88.2 %	(67)	
	No	11.8 %	(9)	
	*Missing*			*1*
Tumour	Pancreas	35.1 %	(27)	
	Liver *(HCC, metastatic CRC)*	54.5 %	(42)	
	Bile duct *(cholangiocarcinoma)*	10.4 %	(8)	
Surgery	Pancreaticoduodenectomy 22.1 % (17)			
	Distal pancreatectomy 10.4 % (8)			
	Total pancreatectomy 2.6 % (2)			
	Major liver resection 42.9 % (33)			
	Minor liver resection 22.1 % (17)			
Smoking	0 %			
Alcohol use	0 %			

### Differences over time regarding the EuroQol-5 Dimensions, EQ-5D-Index and EQ-5D-VAS (perceived health) pre- and postoperatively

The patient's overall reported mobility was assessed as slight problems (preop 16.9 %/ postop 22.1 %). Only 14.3 % graded mobility as a moderate problem postoperatively, and 63.6 % had no problems. Regarding self-care (overall), 24.7 % increased postoperatively from 7.8 % to slight problems. The patients graded moderate problems regarding their usual activity from 11.7 % (preoperative) to 37.7 % (postoperative). The dimension of pain was graded to a greater extent postoperatively, which may be to be expected shortly after major surgery.

In the case of anxiety, the percentage of patients without anxiety increased postoperatively. The group with slight anxiety decreased from 51.9 % (preoperatively) to 40.3 % (postoperatively). The overall EQ-Index score changed from 0.9 to 0.82 (mean), and the EQ-VAS score changed from 73 to 65. Table 2 presents the differences in the Index Score and EQ-VAS between preoperative and postoperative measurements, including the full range of responses (e.g., slight, moderate, severe problems) alongside the dichotomized results shown in suppl material.

A repeated measures multivariate analysis of covariance (MANCOVA) was conducted to examine the relationships between the five dimensions of the EQ-5D scale and the dependent variables (Table 2). The overall mobility and anxiety dimensions did not exhibit significant changes between the preoperative and postoperative assessments. Conversely, the self-care dimension showed a significant decrease over time (*p* = 0.001), as did the dimensions of usual activities and pain (*p* < 0.001). Notable differences were observed in specific tumour groups when comparing preoperative and one-month postoperative assessments. Patients with pancreatic cancer reported fewer problems with activities (*p* = 0.001) and pain (*p* = 0.029) postoperatively. Similarly, in the liver cancer patient group, a higher percentage of men reported no problems with mobility postoperatively (*p* = 0.003). Significant changes were also noted between the preoperative and postoperative assessments in the dimensions of mobility (p = 0.003), self-care (*p* = 0.014), activities (*p* < 0.001), and pain (*p* = 0.018) (Table 2).

**Table 2:** A repeated measures multivariate analysis of covariance (MANCOVA) between the five dimensions of the EQ-5D scale and the dependent variables^a^.

Percent^b^ (95 % CI)	Preoperative	Postoperative	*P*-value	Effect size^f^
Overall^c^				0.561
No problems with MOBILITY	27.4 % (17.3–37.5)	35.6 % (24.2–47.0)	0.172	
No problems with SELF-CARE	11.0 % (3.4–18.5)	28.8 % (18.0–39.6)	0.001^a^	
No problems with USAL ACTIVITY	35.6 % (24.3–47)	74.0 % (63.7–84.2)	>0.001^a^	
No PAIN/DISCOMFORT	57.5 % (45.6–69.5)	82.2 % (73.0–91.4)	>0.001^a^	
No ANXIETY / DEPRESSION	63.0 % (51.2–74.8)	60.3 % (48.7–71.9)	0.605	
Pancreas^d^				0.667
No problems with MOBILITY	20.8 % (3.1–38.6)	29.2 % (8.6–49.7)	0.427	
No problems with SELF-CARE	12.5 % (−4.6–29.6)	25.0 % (2.3–47.7)	0.125	
No problems with USAL ACTIVITY	41.7 % (17.2–66.1)	83.3 % (65.3–100)	0.001^a^	
No PAIN/DISCOMFORT	58.3 % (37.0–79.7)	83.3 % (68.8–97.8)	0.029^a^	
No ANXIETY / DEPRESSION	58.3 % (35.1–81.6)	62.5 % (42.5–82.5)	0.651	
Liver^d^				0.483
No problems with MOBILITY	31.7 % (16.6–46.8)	34.1 % (20.3–48)	0.732	
No problems with SELF-CARE	12.2 % (2.3–22.1)	24.4 % (10.6–38.2)	0.081	
No problems with USAL ACTIVITY	36.6 % (20.6–52.6)	65.9 % (51.0–80.7)	>0.001^a^	
No PAIN/DISCOMFORT	58.5 % (44.3–72.8)	80.5 % (69.3–91.7)	0.012^a^	
No ANXIETY / DEPRESSION	65.9 % (50.4–81.3)	58.5 % (42.4–74.7)	0.273	
Women^e^				0.617
No problems with MOBILITY	37.1 % (21.9–52.4)	28.6 % (11.4–45.7)	0.360	
No problems with SELF-CARE	11.4 % (0–23.7)	25.7 % (10.0–41.5)	0.016^a^	
No problems with USAL ACTIVITY	42.9 % (24.6–61.1)	68.6 % (52.4–84.7)	0.004^a^	
No PAIN/DISCOMFORT	51.4 % (32.8–70.0)	77.1 % (62.0–92.2)	0.005^a^	
No ANXIETY / DEPRESSION	65.7 % (48–83.4)	65.7 % (50.1–81.3)	1.000	
Men^e^				0.627
No problems with MOBILITY	18.4 % (5.0–31.8)	42.1 % (24.8–59.4)	0.003^a^	
No problems with SELF-CARE	10.5 % (0–21.5)	31.6 % (15.7–47.5)	0.014^a^	
No problems with USAL ACTIVITY	28.9 % (13.1–44.8)	78.9 % (64.6–93.3)	>0.001^a^	
No PAIN/DISCOMFORT	63.2 % (46.2–80.1)	86.8 % (76.8–96.9)	0.018^a^	
No ANXIETY / DEPRESSION	60.5 % (45.6–75.5)	55.3 % (39.5–71.1)	0.408	

a The difference is significant at the 5 % level.

b Estimated proportion with Repeatad Measures MANCOVA were all the dimensions are included.

c Adjusted for the covariates: sex, age, education level, marital status, children, tumour and EQ-VAS change.

d Adjusted for covariates: sex, age, education level, marital status, children and EQ-VAS change.

e Adjusted for covariates: age, education level, marital status, children, tumour and EQ-VAS change.

f Effect sizes measured with partial eta squared (η^2^p).

### EQ-5D-VAS (perceived health) and EQ-5D-Index pre- and postoperatively

**Fig. 1:**
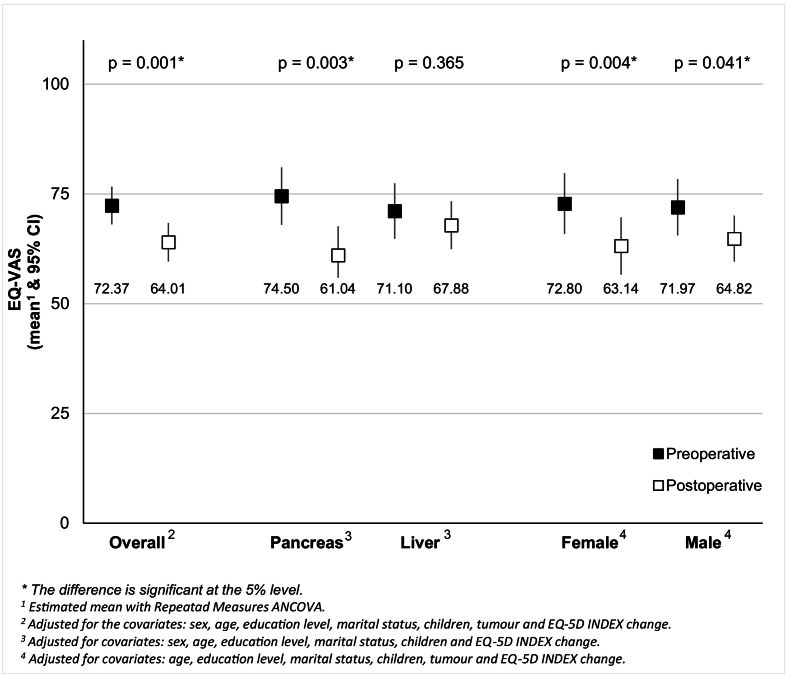
EQ-5D-VAS (perceived health) pre- and postoperatively.

Responses to the EQ-VAS (perceived health) are presented in Fig. 1. The overall EQ-VAS scores, as well as those for the pancreatic group, females, and males, were statistically significantly reduced between preoperative and postoperative measurements (Fig. 1). However, patients with liver cancer showed no change in EQ-VAS scores between preoperative and postoperative measurements (*p* = 0.365).

**Fig. 2:**
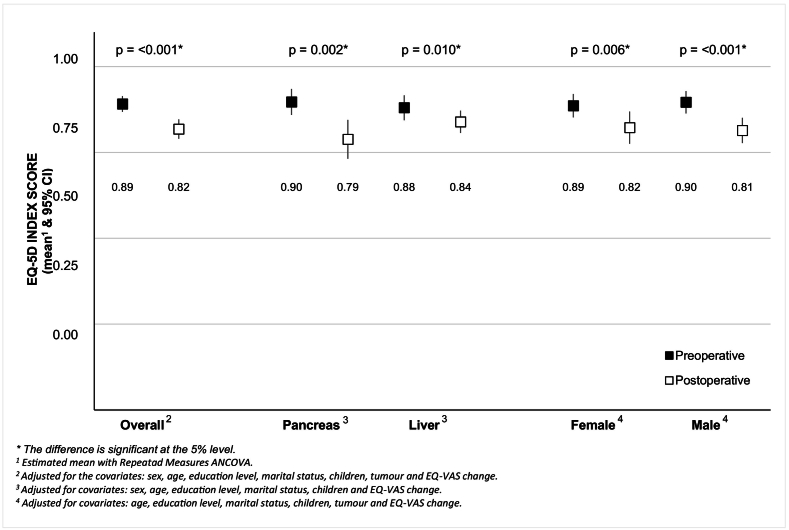
EQ-5D-Index pre- and postoperatively.

The overall EQ-5D-Index was reduced between the pre- and postoperative groups (Fig. 2). The reduction in the EQ-5D-Index was statistically significant in the pancreatic group, liver group, females and males between pre- and postoperative measurements. The results were interpreted as a clinically important difference.

## Discussion

The current study aimed to investigate pre- and postoperative changes in health-related quality of life (HRQoL) in hepatopancreatobiliary (HPB) surgical patients. The intent of the prospective study design was to gather evidence and knowledge about HPB patients' assessed health pre-surgery and post-surgery. This knowledge holds clinical value, enabling healthcare providers to support patients closely upon discharge from the hospital. Generally, health decreased after surgery in both the EQ-5D index and the EQ-5D visual analogue scale (VAS). Previous evidence has demonstrated reduced health for patients with pancreatic cancer [1]. However, the previous evidence is not concordant. A systematic review [9] showed more diverse results for patients with pancreatic cancer. Some included studies did not report any change in general health, while others showed decreased HRQoL. A decrease in health during the first three months postoperatively is common [11,12]. Reduction in health outcomes in patients with liver cancer has been noted in existing evidence [13,14] and a reduction in health outcomes during the first three or four months is common. Our study results showed decreased HRQoL post-surgery. The differences found in this study may lead to improved surgical care, and the results are clinically important for providing the best supportive cancer care.

It is noteworthy that patients with liver cancer showed no change in EQ-5D VAS between pre- and postoperative measurements. Previous evidence has shown generally lower HRQoL in this group of patients [14,17]. To our knowledge, there is sparse evidence on HRQoL in patients with liver cancer who underwent surgery [17], and we have not found any studies that have assessed patients' graded health after surgery.

The overall self-care dimension improved (*p* = 0.001), and the usual activities and pain dimensions (*p*≥ 0.001) also improved over time. The patient's reported mobility was assessed as slight problems preoperative and postoperative, and over half of the patient's graded mobility as no problem one-month postoperatively. Maybe a reason that the majority participate in enhanced recovery programs. Enhanced recovery programs have been shown to improve postoperative outcomes after abdominal surgery [18] and the adherence to the program is high in the clinical context where the study was performed.

Regarding anxiety, the patients who were slightly anxious preoperatively decreased postoperatively. The overall anxiety dimensions in this cohort were not differed between the pre- and postoperative assessments. Previous evidence has shown that anxiety and depression may not differ between patients with and without a history of cancer. Anxiety, depression, and difficulties with concentration have been shown to impact other health-related dimensions [19]. High-quality supportive care for these participating patients may have influenced these anxiety dimension outcomes. Evidence-based nursing care has been shown to significantly improve anxiety, depression, and health outcomes following interventions in patients with liver cancers [20]. Previous evidence shows that psychological symptoms such as anxiety and depression occur in 50 % of patients before diagnosis of pancreatic cancer [21]. A significant percentage of patients with pancreatic cancer experienced depression and anxiety [22], and their caregivers [23]. Multidimensional psychosocial predictive factors and psychological care is of importance [22].

It is important to enhance substantial hope before and after surgery, the fact that the surgery may give patients a chance to survive and improve a sense of control over the situation. Early discharge and being in a home environment have shown to support patients to be more positive [24]. However, there is few studies regarding psychological condition and anxiety after pancreatic and liver surgery, highlighting the need to explore patients' psychological health in the surgical setting [[24], [25], [26]].

The overall self-care dimension significantly changed over time (*p* = 0.001). These results highlight the importance of supportive care after discharge and the person-centred approach in surgical care to support patients' self-care. This approach may be challenging to implement into routine clinical practice, but studies have shown that strategies to reduce patients' self-care issues/burden may include the incorporation of effective interdisciplinary supportive care planning [27,28]. Mobile-system solutions and application-based technology may have also shown positive outcomes of effective self-care support [28,29].

Pain was reported postoperatively, which is not surprising, especially after major surgery. However, it is interesting that men reported fewer problems with mobility postoperatively, while women did not significantly differ in mobility between pre- and postoperative measurements. One speculation may be that mobility is related to patients' perceived pain, which can vary between men and women. Evidence has shown that gender/sex may affect how an individual contextualizes and copes with pain. Pain conditions are more common in women, with a higher prevalence than in men [30]. The clinically important difference is the difference that the patient is able to recognize [31]. Our results, due to postoperative mobility differences between women and men, are interpreted as clinically important and may lead to tailored patient information to stimulate mobility in women postoperatively and adequate pain control.

Differences between specific tumour groups and dimensions in the pre- and postoperative assessments were found. Patients with pancreatic cancer had graded fewer problems with activities and pain postoperatively, which was significant. In the patient group with liver cancer surgery, the results seemed to be similar. Pancreatic cancer is a diagnosis with poor prognosis, and some previous studies have shown that HRQoL is reduced [1,3], but other studies have shown more varied results (van Dijk et al., 2018). Patients often have many symptoms, e.g., pain and fatigue [1,32]. Pain seems to be worse for patients during the first three months after surgery [32,33]. However, even if the surgery is performed, the patients may have an advanced cancer disease that requires oncological treatment that affects the patients' health in different ways in a longer time perspective. However, a review shows that the current evidence is sparsely related to treatments for patients with advanced pancreatic cancer, e.g., lack of reports of patient-centred outcomes [34]. In a comprehensive 10-point action plan, the most important highlight the patient perspective, improve survival and experienced health [35]. Specific attention should be focus on the vulnerable group of older patients. Effective postoperative follow-up may improve patients' well-being and also relief from their symptoms, and telenursing has shown to be effective [36]. More clinical PROM: s should be performed at diagnosis and during cancer treatments trajectory to contribute to the best understand and supportive care in concordance with the patients' needs and to detect unmet needs [[37], [38], [39], [40]]. Clinical implications include tailored patient information. The differences in postoperative mobility between men and women may lead to tailored patient information and adequate pain control. High-quality supportive care can significantly impact anxiety, depression, and overall health outcomes. Implementing a person-centred approach in surgical care can support patients' self-care and improve their overall well-being over a longer follow-up period. The follow-up period in our study is quite short and HRQoL might change over a longer period, such as 3 months or 6 months. Extending the follow-up period would provide a more comprehensive insights into the sustained impact of surgery on patients' HRQoL. A longer duration would allow for the observation of potential long-term benefits or complications that may not be evident within the initial month. This extended follow-up could also help in understanding the trajectory of recovery and adaptation, offering a more robust evaluation of the surgery's effectiveness and its implications for patient care.

### Limitations

This study has several limitations that should be considered when interpreting the results. Firstly, the sample size was relatively small (*n* = 77), which may limit the generalizability of the findings. A small sample size can reduce the statistical power of the study, making it more challenging to detect a true effect if one exists (in subgroup analyses). Additionally, the response rate of 76.2 %, while acceptable, indicates that nearly a quarter of the patients did not provide complete data, potentially introducing response bias. This incomplete data can affect the study's validity and reliability, as the missing responses might differ systematically from those who completed the survey. Furthermore, the limited sample size and response rate could impact the statistical significance of the results, as smaller samples are more prone to variability and may not accurately represent the broader population.

The demographic characteristics of the sample, with over half of participants being 70 years or older, may also influence the outcomes, as older patients might experience different postoperative recovery trajectories compared to younger individuals. In addition, 55.8 % were 70 years or older, nearly all patients are over 60 years (87 %). Furthermore, the study did not account for potential confounding variables such as specific comorbidities, socioeconomic status which could affect health outcomes. This study provides patient-reported data on alcohol consumption and smoking habits. However, the information regarding alcohol and smoking may be subject to recall bias and social desirability bias. These biases could potentially affect the accuracy of the reported information. Furthermore, it is important to note that the study did not include data on weight or Body Mass Index (BMI) and readmission. The absence of this data is due to the lack of availability within the cohort and the absence of ethical approval to access patients' medical records. This limitation restricts our ability to explore the potential impact of readmission and weight/BMI on the outcomes studied. Future research should aim to incorporate more comprehensive data collection methods, including objective measures of weight/BMI, to enhance the robustness of the findings. Another limitation is the short follow-up period postoperatively. This timeframe may not capture the long-term effects of surgery on HRQoL, and further research with extended follow-up is necessary to understand the sustained impact on patients' quality of life.

## Conclusions

The clinically significant results include that mobility, and anxiety did not change between the pre- and one-month postoperative assessments. Men had a higher reported no problems with mobility postoperatively. The differences in mobility postoperatively between women and men are interpreted as important clinical findings. These results suggest that more attention is needed to support patients to ensure equitable and safe postoperative cancer care.

The following are the supplementary data related to this article.

Table 2 Differences of the Index Score and the EQ-VAS between the preoperative and the postoperative measurements. Table 2

## CRediT authorship contribution statement

**Anna Ekström:** Writing – review & editing, Writing – original draft, Visualization, Validation, Methodology, Formal analysis, Data curation. **Victoria Fomichov:** Writing – review & editing, Writing – original draft, Visualization, Software, Methodology, Formal analysis, Data curation. **Bergthor Björnsson:** Writing – review & editing, Visualization, Validation, Supervision, Methodology, Conceptualization. **Carina Wennerholm:** Writing – review & editing, Writing – original draft, Visualization, Validation, Supervision. **Per Sandström:** Writing – review & editing, Visualization, Validation, Supervision, Methodology, Conceptualization. **Jenny Drott:** Writing – review & editing, Writing – original draft, Visualization, Validation, Supervision, Software, Resources, Project administration, Methodology, Investigation, Funding acquisition, Formal analysis, Data curation, Conceptualization.

## Ethics approval

Informed consent was obtained from all individual participants included in the study.

The study was performed in accordance with the ethical principles of the Declaration of Helsinki and approved by the Regional Ethics Review Board (No. 2016/276–31).

## Funding sources

This work was supported by grants from the 10.13039/100010805Medical Research Council of Southeast Sweden (FORSS-862001).

## Declaration of competing interest

The authors declare no competing interests.
